# Effects of Different Hangboard Training Intensities on Finger Grip Strength, *Stamina*, and *Endurance*

**DOI:** 10.3389/fspor.2022.862782

**Published:** 2022-04-12

**Authors:** Marine Devise, Clément Lechaptois, Eric Berton, Laurent Vigouroux

**Affiliations:** ISM, CNRS, Aix-Marseille University, Marseille, France

**Keywords:** climbing, training, force intensity, finger strength, fatigue, stamina, endurance

## Abstract

Climbing-specific training programs on hangboards are often based on dead-hang repetitions, but little is known about the real intensity applied during such effort. The aim of this study was to quantify and compare the effects of different training intensities (maximal, high submaximal, and low submaximal intensities) on the fingers' physiological capabilities using a hangboard fitted with force sensors. In total, 54 experienced climbers (13 women and 41 men) were randomly divided into four groups, with each group following different training intensity programs: maximal strength program performed at 100% of the maximal finger strength (MFS; F100), intermittent repetitions at 80% MFS (F80), intermittent repetitions at 60% MFS (F60), and no specific training (control group). Participants trained on a 12 mm-deep hold, twice a week for 4 weeks. The MFS, *stamina*, and *endurance* levels were evaluated using force data before and after training. Results showed similar values in the control group between pre- and post-tests. A significantly improved MFS was observed in the F100 and F80 groups but not in the F60 group. Significantly higher *stamina* and *endurance* measurements were observed in the F80 and F60 groups but not in the F100 group. These results showed that a 4-week hangboard training enabled increasing MFS, *stamina* and *endurance*, and that different improvements occurred according to the level of training intensity. Interestingly, the different intensities allow improvements in the targeted capacity (e.g., *stamina* for the F80 group) but also in the adjacent physiological capabilities (e.g., MFS for the F80 group).

## Introduction

The introduction of sport climbing at the recent 2021 Olympic Games in Tokyo is the result of a considerable increase in the number of recreational and competitive practitioners and climbing structures. Climbing performance requires a complex combination of physiological, psychological, technical, and tactical resources for successfully climbing a particular route or mastering a boulder problem (Saul et al., 2019). Among such resources, a key factor for performance is the ability to generate finger strength and the ability to limit forearm muscle fatigue (Watts et al., 1996). Climbing indeed generates intense and intermittent isometric contractions of the muscles actioning the hand and the fingers, especially those located in the forearm (Ferguson and Brown, 1997).

During climbing, three physiological forearm parameters have been identified: (i) the capability to exert maximal finger force on holds (Schweizer and Furrer, 2007), (ii) the time to exhaustion (called *stamina* here) which is the capacity to maintain a certain level of high-force intensity before fatigue, i.e., before loss of strength occurs over time (Quaine et al., 2003), and (iii) the level of force intensity that the climber is still able to sustain once he/she is in a state of exhaustion (Vigouroux and Quaine, 2006), i.e., after having experienced the onset of fatigue [known as critical force (Giles et al., 2019) or *endurance* here]. These physiological parameters are peculiar to climbing and are the results of specific physiological phenomena acting at the forearm level. Specifically, forearm ischemia occurs at 45–75% of maximal finger grip force (Barnes, 1980; Macleod et al., 2007) which implies that climbers should develop muscle force capabilities and local anaerobic and aerobic capabilities and also capabilities to limit the effect of the local ischemia on muscle physiology. These determinants make climbing a unique activity in that it involves training principles in fingers, hands, and forearms that are not found in other sports.

To maximize the climbers' physiological capabilities, climbing alone is a good strategy for novices, but not sufficient for more experienced climbers (Hörst, 2008). This is the reason as to why climbing-specific training tools such as hangboards have been developed and are widely used both by the trainers and by the climbers (Hörst, 2008). Hangboards are equipped with holds of various shapes, sizes, widths, and depths. To improve the maximal finger strength, some training techniques propose to work at the maximum intensity by hanging for a short time (e.g., 3 s) with maximal added weight or on the minimum depth edge and repeat it several times (e.g., 3 repetitions with 60 s rest time; López-Rivera and González-Badillo, 2012; Levernier and Laffaye, 2019; Mundry et al., 2021). To enhance *stamina*, some training sessions propose hanging intermittently (e.g., López-Rivera and González-Badillo, 2019; alternating 10 s hanging and 5 s resting), generally with the full body weight, on a hold less than one phalange deep (López-Rivera and González-Badillo, 2019), for a required number of repetitions (Medernach et al., 2015) usually defined to be close to failure during the last one (López-Rivera and González-Badillo, 2019). To work at the *endurance* level, training with more dead-hang repetitions is proposed, but at moderate intensity by reducing body weight using pulleys while hanging and until exhaustion (Giles et al., 2019), in order to induce fatigue and then increase the force level that can be maintained once fatigue is established.

Thus, until now, the intensity of the exercises designed for use on hangboards has been typically judiciously adjusted by modulating the three following parameters: the size of holds, the hanging and rest times and/or the number of repetitions, and adding/subtracting weight while hanging (López-Rivera and González-Badillo, 2012, 2019; Medernach et al., 2015; Levernier and Laffaye, 2019). Thus, training strategies to control intensity consist of manipulating some of these parameters while kipping others equal. For example, Medernach et al. (2015) proposed (in part) to train at body weight by modifying hanging times between 3 and 10 s and number of repetitions between 6 and 10, supervised by the coaches. Nevertheless, such methods could have some limits: first, changing the size (and even the form) of holds implies different positions and surfaces used by fingers and generates different muscle coordination such that the training exercise addresses different synergies instead of only changing the intensity. Second, modulating the time or the number of repetitions to adjust exercise intensity (e.g., hanging less time or less repetitions on the same hold with body weight to decrease the difficulty) may involve a change in the targeted physiological capacities instead of changing only the exercise intensity (e.g., from *stamina* to strength when decreasing the hanging time or the repetitions). Finally, using additional or reducing weight is not convenient as it requires use of harness, rope or loads, and it is hard to set the accurate right level of weight for each repetition.

Nowadays, newly developed instrumented hangboards or single holds provide improvement in feasibility and accuracy, and are a valid and reliable measurement of applied loads with an accuracy in the <1 N range (Anderson, 2018; Michailov et al., 2018; Vigouroux et al., 2018; Feldmann et al., 2021; Marino et al., 2021). These instrumented tools technically allow modulating training exercise intensity thanks to force visual feedback, on the same hold. Though, little is known about how this modulation should be carried out to obtain the best improvements in the various physiological capacities. For example, with a maximal intensity exercise targeting maximal finger strength improvement, we ignore the impact on other physiological parameters (*stamina* and *endurance*). This approach is crucial to clarify as it is consistent with many training programs in other sports that modulate intensity of force exerted during exercise (Bompa and Carrera, 2005; Suchomel et al., 2021). We are also questioning what physiological adaptation processes are involved depending upon the intensity.

Thus, the aim of this study was to quantify the effects of several training intensities on the finger's physiological capabilities with an instrumented hangboard. Three training programs (maximal, high-submaximal intensity, and low-submaximal intensity) performed during 4 weeks were tested. We hypothesized that the improvements in the three physiological parameters (maximal finger strength, *stamina*, and *endurance*) are different depending on the level of force intensity required during the training exercises, i.e., high-intensity training increases maximal strength and low-intensity training increases resistance to fatigue. We also hypothesized that the amount of benefit in one physiological parameter is dependent on its baseline level (whether maximal finger strength, *stamina*, or *endurance*).

## Materials and Methods

### Participants

In total, 54 climbers were tested (13 women and 41 men, 25.0 ± 6.2 years old, 173.6 ± 8.3 cm, 64.2 ± 8.7 kg). They were advanced or elite climbers according to IRCRA (International Rock Climbing Research Association) scale (Draper et al., 2015), mostly lead rock practitioners (see Table 1 for red-point grade). They were randomized into four different training protocols, each following different training intensity programs or no specific training (control group, CT). Participants had no hand or upper extremity injuries in the 6 months prior to the test. They were informed of the risks and benefits of the research protocol and signed a consent form. Protocol has been validated by the sport science national ethics committee.

**Table 1:** Descriptive characteristics of the participants of each group (mean ± SD).

	**F60 (*n* = 14)**	**F80 (*n* = 14)**	**F100 (*n* = 14)**	**CT (*n* = 12)**	***p*-value**
Age (y)	23.8 ± 4.3	23.4 ± 5.0	23.3 ± 4.5	28.8 ± 9.4	0.08
Height (cm)	171.1 ± 8.9	174.4 ± 9.7	174.1 ± 7.0	175.0 ± 7.5	0.62
Body mass before training (kg)	62.6 ± 7.5	64.1 ± 9.2	63.7 ± 7.9	67.0 ± 10.8	0.63
Body mass after training (kg)	62.6 ± 7.9	64.3 ± 9.1	63.5 ± 7.6	67.4 ± 10.9	0.57
“On-sight” performance (au)	18.3 ± 2.7	17.6 ± 3.5	17.2 ± 2.6	18.1 ± 3.6	0.83
“Redpoint” performance (au)	21.7 ± 3.7	20.9 ± 3.3	20.5 ± 3.3	21.2 ± 3.8	0.82

“*On-sight” performance means climbing a sport route at the first attempt without any information about it; “Redpoint” performance means climbing a sport route after inspecting and practicing it. Both performances represent the most difficult grade achieved in the past 6 months and are converted to the IRCRA scale. p-values represent results of the one-way ANOVA comparing the four tested groups*.

### Procedures

**Figure 1:**
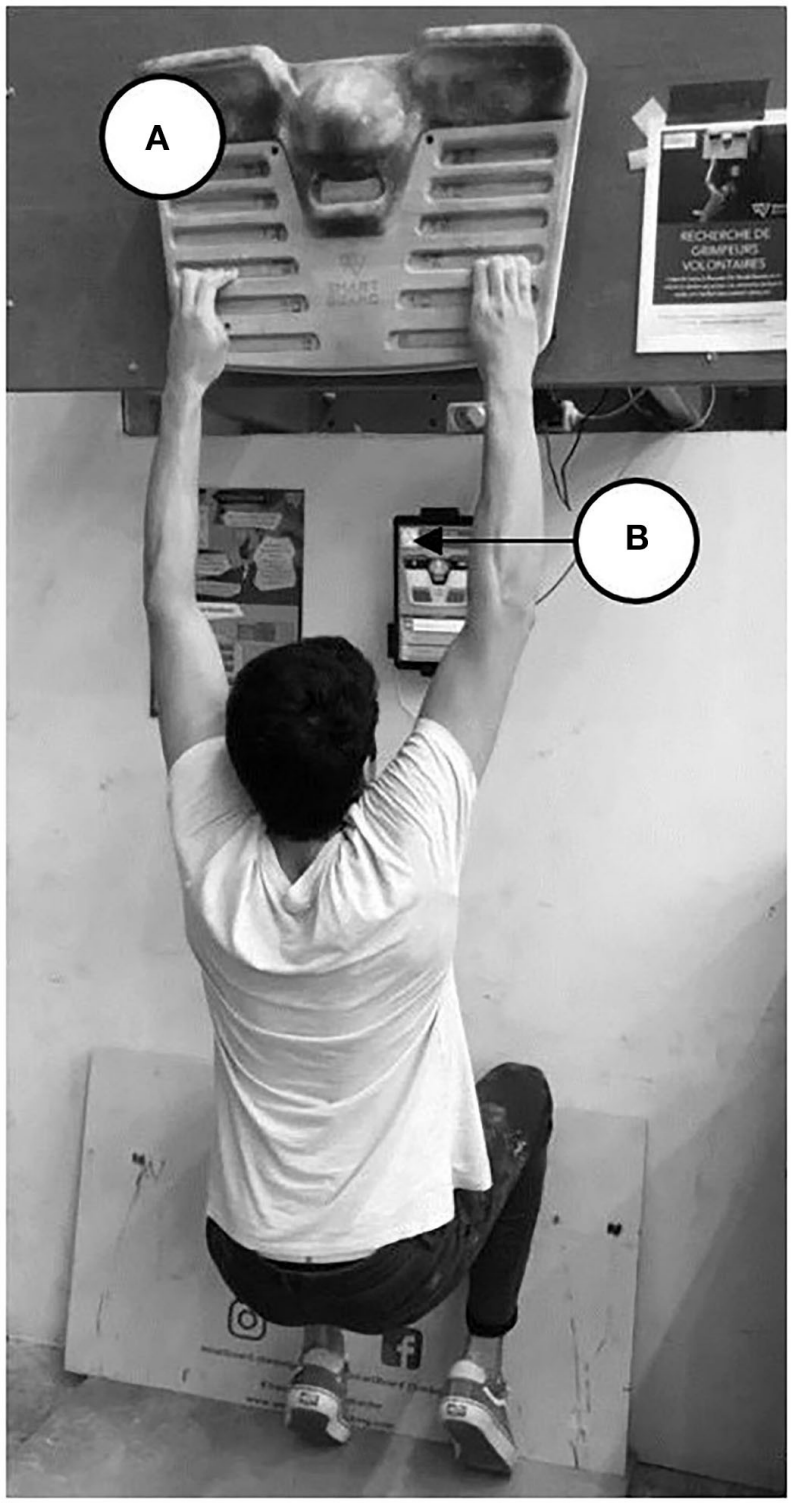
Force-time curve during the 24 repetitions of the fatigue test of one representative participant. *Stamina* (represented by the horizontal arrow) is the capacity to maintain the 80% of maximal finger strength (MFS) threshold. *Endurance* is the percentage of force the participant is able to perform after the onset of fatigue (represented by the dotted black line). On this test, the participant performed 633 N for MFS, 45.2% for *stamina* and 70.3% for *endurance*.

The participants first performed some initial tests (week 1) consisting of an assessment of maximal finger strength (MFS), *stamina*, and *endurance* on a hangboard fitted with strain gauges (SmartBoard, Peypin d'Aigues, France). This hangboard (Figure 1) provides real-time feedback about the vertical force applied on it allowing precise modulation of the force intensity (1 N accuracy). Data concerning the force applied to the holds were recorded and analyzed using the SmartBoard app (50 Hz). For the following 4 weeks (weeks 2–5), they followed one of the three training protocols [except for the CT group (*n* = 12)] on the instrumented hangboard with 2 sessions per week, with at least 1 day rest between sessions. Participants were instructed to continue their climbing activity normally outside of the study without increasing or decreasing their current practice. Post-training tests, identical to the initial ones, were performed at week 6 in all the groups. In order to avoid fatigue effects, the participants were not allowed to climb the day before the initial and post-tests.

### Test Sessions (Weeks 1 and 6)

#### Strength Test

After a 30 min warm-up and familiarization with the SmartBoard, consisting of muscular awakening, easy traverses and specific exercises on the SmartBoard with increasing intensities, climbers had to exert the maximum force with one hand on a 12 mm hold for 6 s (climbers were weighted when needed to perform a force intensity higher than their body weight). The type of grip (slope, half-crimp, and full crimp) was self-selected and it was required that participants used the same grip throughout the experiment. Two trials were tested on each hand, and the best was selected. The sum of the maximum forces exerted with the right and left hands was directly displayed on the interface and was considered as the participant's initial maximal finger strength (MFSi) at week 1 and post-MFS (MFSp) at week 6.

#### Fatigue Test

**Figure 2:**
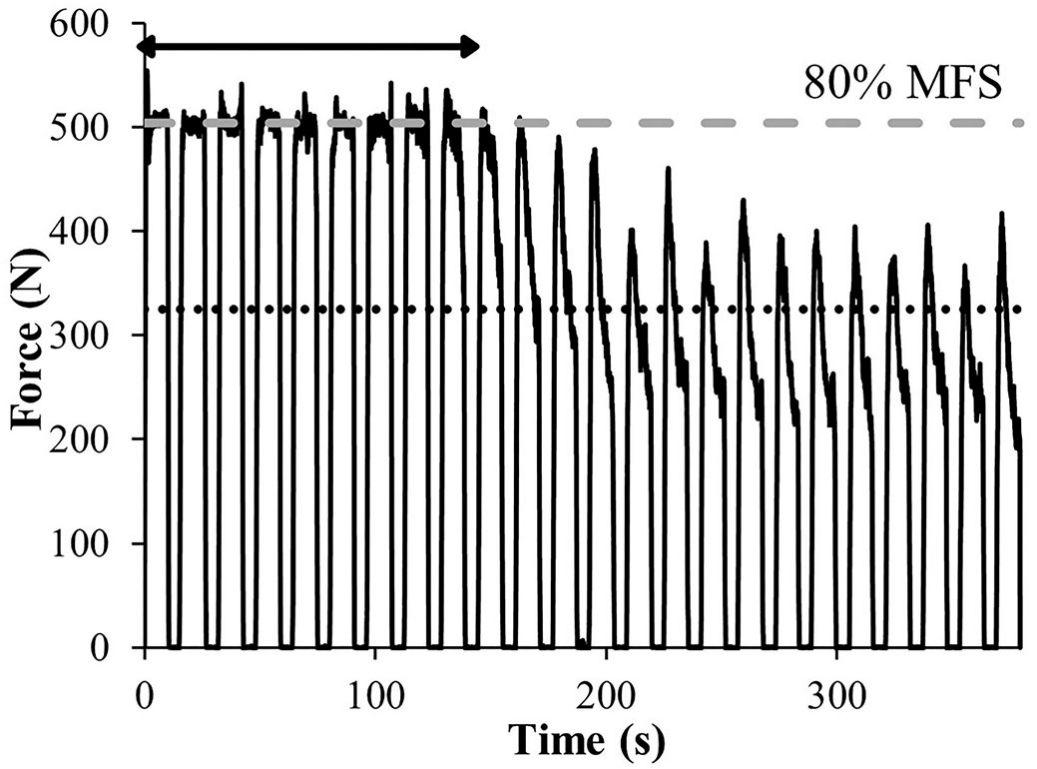
Illustration of one participant during the fatigue test performed on the SmartBoard **(A)**, on the 12 mm hold. The feedback of the force level was displayed on the tablet **(B)** so that the participant can adjust the level of force intensity required during the test/training by using his feet on the ground.

On a 12 mm-width ledge, participants exerted 80% MFSi by alternating a hanging phase of 10 s and a rest phase of 6 s during 24 repetitions. The 80% level was controlled by the visual force feedback and carefully adjusted by off-loading with feet on the ground or conversely using additional ballast. The fatigue test reproduced the one performed in Vigouroux and Quaine (2006): when the subjects were not able to maintain the required 80% MFSi, they were required to continue the exercise and exert the maximum level of force they are able. Generally, this last part was performed by off-loading at the minimum possible the body weight with feet on the ground as illustrated in Figure 1. The recorded fatigue kinetic (Figure 2) allows evaluating the percentage of *stamina* (determined as the capacity to maintain the required 80% for the overall duration of the test) and the percentage of *endurance* (determined as the level of force intensity that the climber is able to perform when he is exhausted in comparison to the initial level of force), directly displayed by the app.

### Training Sessions (Weeks 2–5)

Each training protocol used the same 12 mm hold. For this study, three different training programs (F60, F80, and F100) were tested. These trainings are detailed later and were determined (i) from literature to develop maximal force intensity (F100) and (ii) to induce fatigue (F60 and F80) with different force intensities but similar overall loads.

#### F60 Protocol

Participants (*n* = 14) exerted efforts representing 60% MFSi by alternating a 10 s-hang phase with the two hands and a 6 s-rest phase, 24 times. The 60% level was controlled throughout the protocol by the visual force feedback and adjusted carefully by off-loading with feet on the ground or conversely using additional ballast. Once fatigue occurred, participants were required to continue the exercise and exert the maximum level of force they are able until the 24th repetition. Two sets were performed, separated by a 6-min recovery period.

#### F80 Protocol

Participants (*n* = 14) exerted 80% MFSi, by alternating a 10 s-hang phase with the two hands (with or without feet on the ground, weighted if 80% MFSi > body weight) and a 6 s-rest phase, for a maximum of 12 times or, once participants were no longer able to exercise 70% MFSi during the hanging phase, the sets was stopped. Three sets were performed, with 8 min of recovery time between each.

#### F100 Protocol

This protocol is based on the Levernier and Laffaye protocol (Levernier and Laffaye, 2019). Climbers (*n* = 14) applied their maximum force with the right hand, then with the left hand, for 6 s each, alternating grip types (slope or half-crimp). If the climbers were able to hang with one hand, they were sufficiently weighted so that they were able to exert their maximal force. Two sets of 6 hangs with each hand were performed every 3 min, with a 5 min-recovery time between sets.

### Statistical Analysis

Data are reported as mean ± SD. One-way ANOVA was performed to evaluate differences of the descriptive characteristics between the groups. The effects of training intensity on MFS, *stamina*, and *endurance*, were assessed by comparing the CT, F60, F80, and F100 groups using a two-factor repeated measure ANOVA (training × group), with Tukey *post-hoc* analysis when ANOVAs were significant. In addition, effect sizes (eta squared, η^2^) were calculated. To evaluate the relationship between initial levels of MFS, *stamina*, and *endurance* and their benefits after training, Pearson test correlations were assessed. Significance level was set at *p* < 0.05. Statistical tests were processed using STATISTICA software (version 6, StatSoft, Inc.).

## Results

The anthropometric data and climbing abilities are summarized in Table 1. No statistical differences between groups were observed for all characteristics (*p* > 0.05).

### Maximal Finger Strength

**Figure 3:**
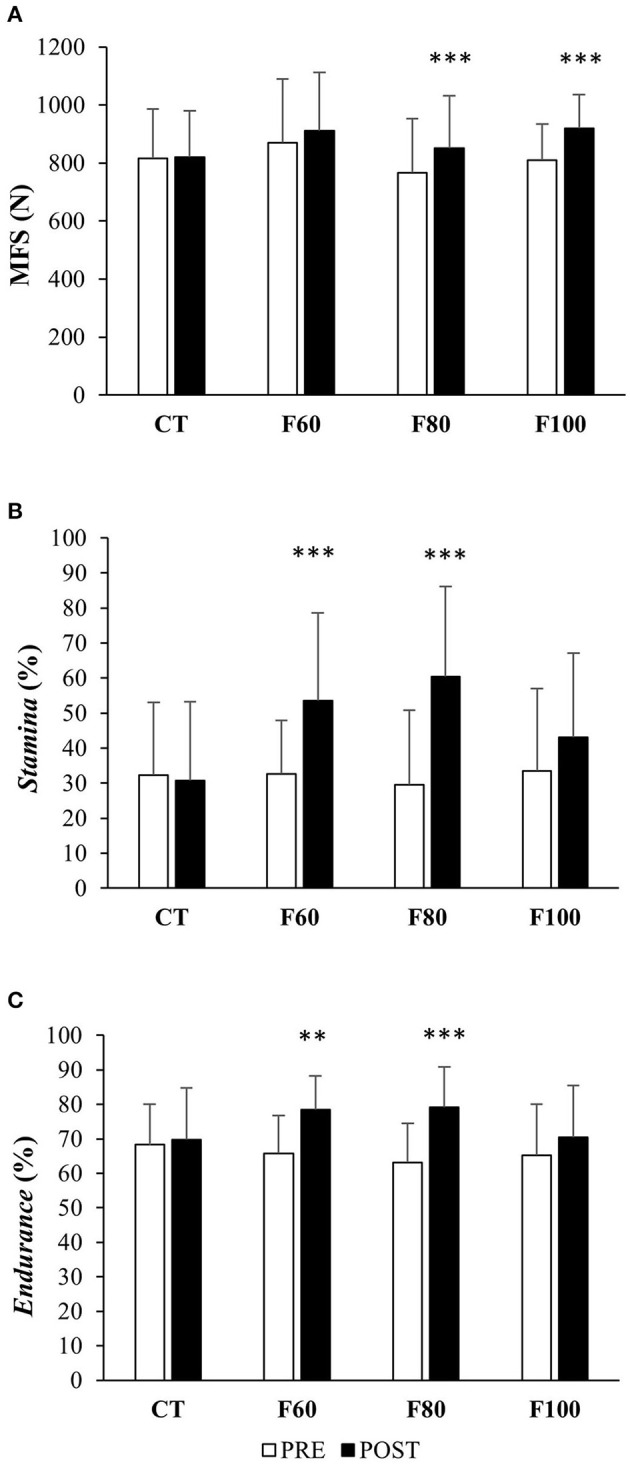
Mean values (± standard types) of **(A)** maximal finger strength (MFS in N), **(B)** *stamina* (in %), and **(C)** *endurance* level (in %) in each group (CT, F60, F80, and F100) pre- (white bars) and post- (black bars) training. Significance differences were shown (**p < 0.01 and ***p < 0.001) relative to pre-training.

Maximal finger strength results before and after training are presented in Figure 3A. Statistical analysis did not show any group effect on MFS [*F*_(3, 50)_ = 0.7; η^2^ = 0.039; *p* = 0.56]. A significant training effect was observed [*F*_(1, 50)_ = 68.9; η^2^ = 0.032; *p* < 0.001] showing that MFS was greater after training than before. Significant interaction [*F*_(3, 50)_ = 10.3; η^2^ = 0.014; *p* < 0.001] showed that the increase in MFS was different by group (+0.7 ± 5.0%; +5.9 ± 8.2%; +12.4 ± 8.4% and +14.3 ± 8.8% for the CT, F60, F80, and F100 groups, respectively). *Post-hoc* tests revealed that MFS was significantly different in the F80 and F100 groups (*p* < 0.001) before (767 ± 186 N and 810 ± 125 N for the F80 and F100 groups, respectively) and after training (852 ± 178 N and 920 ± 116 N for the F80 and F100 groups, respectively) but not in the CT and F60 groups (before: 816 ± 170 N and 869 ± 221 N, respectively; after: 820 ± 160 N and 912 ± 201 N, respectively; *p* > 0.05).

**Figure 4:**
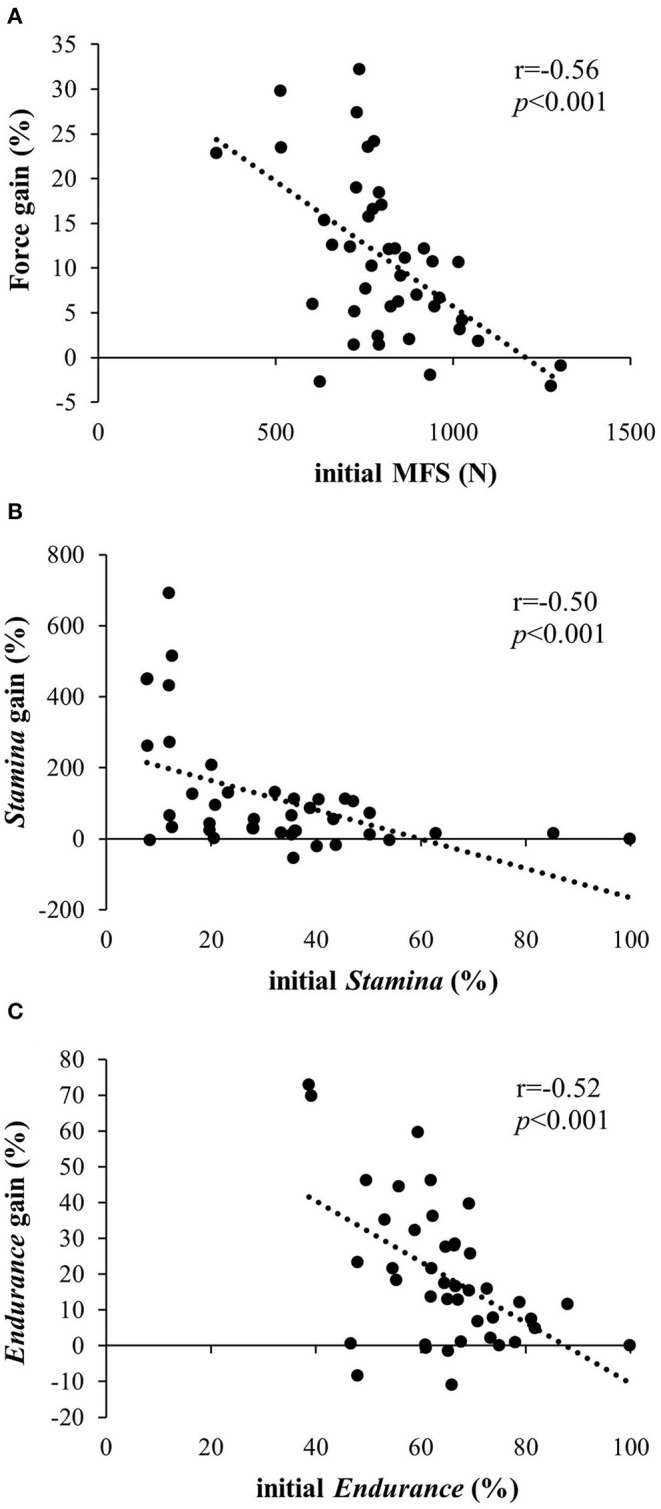
Post-training gain (in %) according to the initial **(A)** MFS (in N), **(B)** *stamina* (in %), and **(C)** *endurance* (in %) for training group subjects (F60, F80, and F100).

When merging the training groups (F60, F80, and F100 groups), a significant but moderate negative correlation (*r* = −0.56; *p* < 0.001) was found between the initial MFS and the force gain during the strength test after training (Figure 4A; *r*^2^ = 0.32).

### Stamina

*Stamina* percentages realized before and after training are presented in Figure 3B. Statistical analysis did not show any group effect on *stamina* [*F*_(3, 50)_ = 1.1; η^2^ = 0.051; *p* = 0.37]. A significant training effect was observed [*F*_(1, 50)_ = 36.3; η^2^ = 0.106; *p* < 0.001] showing that *stamina* was higher after training than before. Significant interaction [*F*_(3, 50)_ = 7.8; η^2^ = 0.071; *p* < 0.001] showed that the increase in *stamina* was different by group. *Post-hoc* tests revealed that *stamina* was significantly different in the F60 and F80 groups (*p* < 0.01) before (32.6 ± 15.3% and 29.5 ± 21.3% for the F60 and F80 groups, respectively) and after training (53.5 ± 25.1% and 60.4 ± 25.7% for the F60 and F80 groups, respectively) but not in the CT and F100 groups (before: 32.4 ± 20.7% and 33.5 ± 23.5%, respectively; after: 30.8 ± 22.5% and 43.1 ± 24%, respectively; *p* > 0.05).

Moreover, when merging the training groups, a significant but moderate negative correlation (*r* = −0.50; *p* < 0.001) was found between the initial *stamina* level and *stamina* benefits during the fatigue test after training (Figure 4B; *r*^2^ = 0.25).

### Endurance

*Endurance* levels realized that before and after training are presented in Figure 3C. Statistical analysis did not show any group effect on *endurance* [*F*_(3, 50)_ = 0.4; η^2^ = 0.019; *p* = 0.77]. A significant training effect was observed [*F*_(1, 50)_ = 50.2; η^2^ = 0.117; *p* < 0.001] showing that *endurance* was higher after training than before. Significant interaction [*F*_(3, 50)_ = 7.1; η^2^ = 0.053; *p* < 0.001] showed that increase in *endurance* was different by the group. *Post-hoc* tests revealed that *endurance* was significantly different in the F60 and F80 groups (*p* < 0.001) before (65.7 ± 11% and 63.2 ± 11.3% for the F60 and F80 groups, respectively) and after training (78.5 ± 9.6% and 79.2 ± 11.5% for the F60 and F80 groups, respectively) but not in the CT and F100 groups (before: 68.3 ± 11.7% and 65.3 ± 14.8%, respectively; after: 69.7 ± 14.9% and 70.4 ± 15%; *p* > 0.05).

Moreover, when merging the training groups, a significant but moderate negative correlation (*r* = −0.52; *p* < 0.001) was found between the initial *endurance* level and *endurance* benefits during the fatigue test after training (Figure 4C; *r*^2^ = 0.27).

## Discussion

This study aimed to investigate the effects of different training programs performed at different intensities on the finger muscle capabilities (MFS, *stamina*, and *endurance*). Results suggest that a 4-week hangboard training program is a powerful method for increasing MFS, *stamina*, and *endurance* levels in the climbers, and also that improvements in the finger muscle capabilities depending on the intensity level of the training exercise. Similar values in the control group between both pre- and post-tests showed that increases observed in other training groups are not attributed to a familiarization effect with the tests nor to other concomitant activities.

The results for initial MFS levels were in the range of those measured in the previous studies (Quaine et al., 2011), although higher MFS values were observed by Medernach et al. (2015), Levernier and Laffaye (2019), and López-Rivera and González-Badillo (2019), while lower values were observed by Amca et al. (2012) and Fanchini et al. (2013). These variations may have several explanations: (i) difference in IRCRA level of climbers across studies considering MFS and IRCRA level are positively correlated (i.e., MFS is higher in elite climbers than in novices; Grant et al., 1996; Baláš et al., 2012), (ii) the “climbing style” since “boulderers” have higher initial MFS values than “lead rock practitioners” (Fanchini et al., 2013), and (iii) difference in grip depth used to perform the force test for the reason that the deeper the grip, the greater the force applied (Amca et al., 2012).

Our results showed that the MFS levels were improved with the F100 and F80 groups, being considered as high-intensity training (70–100% MFS). The increases are probably not due to hypertrophy in the forearms (Shimose et al., 2011; España-Romero and Watts, 2012) but to neural adaptation processes during the first weeks of training (López-Rivera and González-Badillo, 2012), allowing a better capacity to recruit motor units on a given movement and/or an increased discharge rate of individual motoneurons (Škarabot et al., 2021) as well as a better anaerobic capacity in the forearms, in order to generate a major muscle activation (Pitcher and Miles, 1997). MFS also increased in similar proportions for the F80 group whose training represents intermittent exercise with a submaximal load-generating fatigue. In the forearms, this exercise at these intensities generates local ischemia stimulus, through reduced blood flow and lactate accumulation (Saeterbakken et al., 2020). Thus, F80 represents a combination of high-mechanical tension and medium metabolic stress that may be effective to increase muscle strength (Duchateau et al., 2021).

On the other hand, a load of lower intensity (60% of initial MFS for the F60 group) did not seem to have sufficient force intensity to obtain large MFS improvements even if a trend is emerging (+5.9 ± 8.2%; *p* = 0.07). A longer training time and/or a higher training frequency could allow for the higher strength gain, as in the López-Rivera and González-Badillo (2012) study where MFS increased by an average of 0.5% between the 4th and 8th week of training.

Our results showed a greater increase in MFS than those observed in the previous studies (Levernier and Laffaye, 2019) where improvements ranged from +5% (Medernach et al., 2015) to +9.6% (López-Rivera and González-Badillo, 2012). This discrepancy may be related to the lower initial MFS level of our climbers compared with those in the aforementioned studies, since a significant negative correlation (*r* = −0.56; *p* < 0.001) was observed between the MFS gain percentage (when merging the trainings) and the initial MFS (Figure 4), i.e., the higher a subject's initial MFS, the lower the strength gain after training. However, it should be noted that variability is high and only explains about one-third of the results (*r*^2^ = 0.32). Thus, further studies should be conducted to explore the different factors conditioning strength gain.

The F60 and F80 groups significantly increased *stamina* after training, from 32.6 ± 15.3% to 53.5 ± 25.1% and from 29.5 ± 21.3% to 60.4 ± 25.7%, respectively. Thus, by adjusting the force amplitude realized during trainings at a submaximal level (80 and 60%) is an efficient way to improving resistance to fatigue. Improvements in *stamina* with intermittent type training are in agreement with the literature (Medernach et al., 2015; López-Rivera and González-Badillo, 2019), where repetition training on a hangboard showed between 25 and 34% increase in *stamina* after 4 training weeks. It is worth noting that in these articles, the training control was performed based on a time measurement and the intensity based on a grip width control. Observed increase in *stamina* in our study is probably because of an improved aerobic metabolism thanks to the increase in glycogen and phosphagen storage capacities (Bertuzzi et al., 2007). It can also be attributed to a better limitation of local ischemia effects in the forearms which improve supply, irrigation, and consumption of oxygen in the muscle, increasing oxidative capacities of the skeletal muscle (Ferguson and Brown, 1997; Fryer et al., 2016). In addition, the faster lactate shuttle and enhanced glycolytic activity allow for greater effectiveness in managing submaximal loads thanks to an improved muscle recruitment pattern.

Concerning the F80 group, an additional assumption may explain *stamina* increase. By increasing his/her MFS after training, the force applied by the participant to perform the fatigue test becomes <80% of his/her post-MFS value and therefore represents a lower intensity during this exercise, even though his/her absolute value (in Newtons) remains the same. Thus, fewer motor units need to be activated for the same load and there is a potential to recruit a greater number of non-fatigued motor units. This delays the involvement of type II fibers as well as lactate accumulation (Hickson et al., 1988; Marcinik et al., 1991), allowing for energy conservation and a longer time to exhaustion. Higher *stamina* improvement with F80 can thus be explained by the combination of improved aerobic/anaerobic metabolisms and improved MFS.

The F100 group did not show significant increase in *stamina* after 4 training weeks. This is in accordance with our hypothesis since F100 training does not generate fatigue and thus does not result in the fatigue adaptation. However, it is possible that this increase could be greater and becomes significant with longer training duration, as in the López-Rivera and González-Badillo study (López-Rivera and González-Badillo, 2019) in which *stamina*, with a purely strength-targeted method, increases insignificantly by 10% after 4 training weeks but increases significantly by 34% after 8 weeks.

*Endurance* level increased in the F60 and F80 groups, from 65.7 ± 11% to 78.5 ± 9.6% and from 63.2 ± 11.3% to 79.2 ± 11.5%, respectively. On the contrary, *endurance* was not improved by training in the F100 group. As for *stamina*, intermittent training (F60 and F80) generates greater fatigue accumulation than maximum intensity training (F100) and therefore reduces the ability to maintain the effort required throughout the session. Intermittent exercise thus provokes a reduction in the short-term strength of type II muscle fibers, caused by the rapid consumption of energy inputs, and type I muscle fibers as a consequence of hypoxia (Pitcher and Miles, 1997). Nevertheless, this exercise type allows for a better tolerance to fatigue than the F100 group. It also allows for the development of aerobic capacity through a faster reoxygenation in forearms (better vasodilatation) during rest phases (Ferguson and Brown, 1997) as well as a better removal of the muscular metabolites. This promotes adaptation of muscular capacities, limiting fatigue effects to maintain higher intensity strength once fatigue has set in.

Some limitations should be acknowledged. First, the 4-week training interval could be considered as short in comparison to non-climbing specific studies on force development which claim effects after 8 weeks (Morris et al., 2022). In total, 4-week duration is thus not long enough to draw conclusions about middle- and long-term training effects, and further studies are needed. Nevertheless, studies addressing to specific climbing training revealed that a 4-week plan based on finger flexor muscles were sufficient to increase strength in the elite climbers (Medernach et al., 2015; Levernier and Laffaye, 2019). Second, participants did not have the same time practice of climbing activity outside the experiment (according to their own usual practice). A part of the results variability can thus be attributed to this various time of climbing practice and mostly to mixed population (advanced to elite climbers, men, and women, etc.). Third, we chose to base the intensity training on the first session instead of testing climbers before each training session, to not influence the effect trainings. But, it is important to keep in mind that intensity (of F60 and F80 groups) may change over days/weeks because of fatigue in that moment. Finally, the effects of different intensity resistance training on angiogenesis, muscle oxygenation kinetics, and muscle oxidative capacity have not been measured in this study and remain highly speculative as to which physiological pathway-enhanced finger capabilities in the training groups. Additional studies are clearly necessary to investigate the physiological phenomenon under training processes by more measurements such as electromyography, lactatemia, or blood flow (with near infrared spectroscopy) to confirm our physiological assumptions. A last consideration is that the use of a force-cell hangboard enables to compute scores based on force-time data (MFS in Newtons; *stamina* and *endurance* in percentage). This approach is in accordance with the previous laboratory studies (Vigouroux and Quaine, 2006; Giles et al., 2019) but differs from others which focused on evaluation using hanging time (Medernach et al., 2015; López-Rivera and González-Badillo, 2019). Comparison of current results with the literature should be interpreted carefully.

Overall, this study showed that, after 4 weeks of hangboard training, maximal finger strength, *stamina*, and *endurance* increase, that different improvements occurred according to the training intensity levels, and that different training levels allow improvements in the targeted capacities (e.g., *stamina* with F80) but also in the adjacent capabilities (e.g., MFS with F80). Such trainings could be useful to quickly improve the climber's capacities, just before a competition for example. The F100 training improves MFS without reaching physiological exhaustion during the sets allowing to complete it by other works on route or boulder with minimal quality loss in comparison with the F60 and F80 trainings. The F60 training allows benefits with low-force intensity, therefore, it may be suitable for the climbers concerned about a risk of injury or wishing to return to training after a long period of inactivity (Peters, 2001). The F80 training promotes simultaneous enhancement of each physiological parameter, especially useful for a versatile climbing practice. However, these enhancements were a function of the initial level of our climbers as shown by the significant results of regressions in MFS, *stamina*, and *endurance*, i.e., the higher a subject's initial level in one of the physiological parameters, the lower the benefit after training. Presumably, the improvement will be smaller in a group of elite athletes, for example. Further research is thus needed to determine which improvements could be expected in each initial level. As well, it would have been interesting to evaluate a climbing-specific outcome parameter, as IRCRA level enhancement, in order to observe the potential consequences of hangboard training when climbing a harder route/boulder. Nevertheless, since climbing performance is multi-factorial, it remains highly difficult to investigate the relationship between the reported physiological improvements and athletes' performance during climbing (MacLeod, 2009).

## Data Availability Statement

The raw data supporting the conclusions of this article will be made available by the authors, without undue reservation.

## Ethics Statement

The studies involving human participants were reviewed and approved by the Sport Science National Ethics Committee (CERSTAPS: IRB00012476-2020-19-11-69). The patients/participants provided their written informed consent to participate in this study.

## Author Contributions

LV and CL concepted the work and wrote the initial draft. CL and MD carried out the experiments. MD, EB, and LV wrote the manuscript. All authors approved the final manuscript.

## Funding

This research was supported by the National Institute of Sport, Expertise, and Performance (INSEP, France).

## Conflict of Interest

LV and CL report their involvement in the development of SmartBoard in “ScienceForClimbing (SFC)” firm, and their current position as scientific advisors. SFC was not involved in any aspect of this research. The remaining authors declare that the research was conducted in the absence of any commercial or financial relationships that could be construed as a potential conflict of interest.

## Publisher's Note

All claims expressed in this article are solely those of the authors and do not necessarily represent those of their affiliated organizations, or those of the publisher, the editors and the reviewers. Any product that may be evaluated in this article, or claim that may be made by its manufacturer, is not guaranteed or endorsed by the publisher.
